# Fellow eye data for intraocular lens calculation in eyes undergoing combined phacovitrectomy

**DOI:** 10.1111/aos.16741

**Published:** 2024-07-12

**Authors:** Klemens Paul Kaiser, Julian Bucur, Tyll Jandewerth, Thomas Kohnen, Christoph Lwowski

**Affiliations:** ^1^ Department of Ophthalmology Goethe University Frankfurt am Main Germany

**Keywords:** fellow eye, IOL calculation, phacovitrectomy, retinal detachment

## Abstract

**Purpose:**

To evaluate whether the intraocular lens (IOL) calculation of the fellow eye (FE) can be used in eyes undergoing combined phacovitrectomy.

**Methods:**

In this retrospective, consecutive case series, we enrolled patients who underwent combined phacovitrectomy with silicone oil removal and IOL implantation at the Goethe‐University. Preoperative examinations included biometry (IOLMaster 700; Carl Zeiss). We used the IOL calculation of the FE (FE group) to calculate the prediction error compared with the IOL calculation using only the axial length (AL) of the FE (AL‐FE group), as well as using the AL of the operated eye (OE group) in addition to the measurable biometric parameters. IOL calculation was performed using the Barrett Universal II formula. We compared the mean (MAE) and median absolute prediction error (MedAE) with each other. Furthermore, the number of eyes with ±0.50, ±1.00 and ±2.00 dioptres (D) deviation from the target refraction was compared.

**Results:**

In total, 79 eyes of 79 patients were included. MedAE was lowest in the OE group (0.41 D), followed by FE group (1.00 D) and AL‐FE group (1.02 D). Comparison between the AL‐FE and FE groups showed no statistically significant difference (*p* = 0.712). Comparing eyes within ±0.50 D of the target refraction, the OE group (63.3%) performed best, followed by the AL‐FE group (27.8%) and the FE group (26.6%).

**Conclusion:**

Our results indicate no clinically relevant difference between using the IOL calculation of the FE versus using only the AL of the FE in addition to the measurable parameters for the IOL calculation. A two‐step procedure should always be strived for.

## 1 INTRODUCTION

With an incidence of approximately one in 10 000 people per year, rhegmatogenous retinal detachment (RD) is among the main emergency indications in ophthalmology which can cause moderate to severe visual impairment (Haimann et al., 1982). Pars plana vitrectomy (PPV) with vitreous body replacement by oil, gas or air has been established as one of the most effective procedures with a satisfying postoperative anatomical success rate of roughly 90% (Sultan et al., 2020).

When planning the surgical management of eyes with RD, the surgeon needs to consider pre‐existing lens opacification or cataracts to ensure clear visualization during surgery. In addition, PPV leads to cataract progression, and subsequent cataract surgery may be necessary within 1 year after initial vitrectomy (Benson et al., 2021). Recent advances in surgical technology have enabled the ophthalmic surgeon to provide combined management of both cataract and retinal pathology. Combined vitrectomy with phacoemulsification and implantation of an intraocular lens (IOL) has been shown to decrease visual rehabilitation time in patients with early significant cataracts (Villegas et al., 2014). Combined phacovitrectomy is associated with a significant reduction in overall healthcare costs, which may become more and more important in times of healthcare reform (Port et al., 2021; Villegas et al., 2014). Furthermore, it can be assumed that combined surgery reduces the ecological footprint.

In cases of macula‐involving RD, even modern ocular biometry devices as well as the traditional A‐scan can have difficulties measuring the axial length (AL) (Liu et al., 2022). This can also be the case in proliferative vitreoretinopathy, diabetic eyes, vitreous haemorrhage or pronounced vitreous opacity, for instance. In such cases, the IOL calculation of the fellow eye (FE) can be used, since most patients have similar ocular biometry comparing both eyes (Hoffer, 1980; Hoffmann et al., 2022; Liu et al., 2022).

Both ultrasound A‐scan measurements and optical biometry are used to measure the axial length (AL) of the eye. In eyes with vitreous replacement with silicone oil (SO), there are specific differences between these two methods, particularly with regard to the speed of sound and the refractive index. Ultrasound A‐scan measurements are based on the speed of sound in the eye. In a normal eye, the velocity through the vitreous is approximately 1532 m/s (Hoffer, 1994). In an eye filled with SO, this speed changes. Depending on the density and type of SO used, the speed of sound in the SO is around 1000 m/s (Hoffer, 1994). This means that the ultrasonic waves travel more slowly through the silicone oil, which can lead to measurement errors if the correct speed of sound is not taken into account. If the altered speed of sound in the SO is not set correctly, the AL of the eye can be measured incorrectly, which consequently leads to inaccurate results. On the other hand, optical biometry uses light and is based on the refractive index of the media in the eye. The refractive index of the vitreous is roughly 1.336 (Murphy & Howland, 1987). The refractive index of SO is higher and varies depending on the viscosity of the SO used, typically between 1.40 and 1.45 (Stefánsson et al., 1988). As optical biometry takes into account the refractive index of the medium, it can be influenced by the changing optical properties of the SO. It is important to set the correct refractive index to ensure accurate measurements. If the refractive index of the SO is not taken into account, this can lead to errors in the measurement of the AL. Thus, both methods must be adjusted to account for the altered physical properties of the SO in the eye to ensure accurate measurements of the AL of the eye.

In a recently published study, we investigated whether the AL of the FE in combination with the measurable biometric parameters of the affected eye can be used for IOL calculation. In this study, we showed that the use of the AL of the FE leads to a clinically and statistically significant reduction of the IOL power predictability and a two‐stage procedure with measurement of the AL after reattachment of the retina is recommended for the best possible IOL outcome (Lwowski et al., 2023). Nevertheless, in some cases, combined surgery is inevitable, for example, when complex ocular pathology or a significant cataract is present and clear visualization during surgery is necessary.

In the present study, we aimed to find out whether the entire IOL calculation of the FE with all its biometric results can be used in eyes undergoing combined phacovitrectomy or whether the accuracy is greater using only the AL of the FE in addition to measurable biometric parameters of the affected eye for the IOL calculation.

## 2 METHODS

### 2.1 Study design

In this retrospective, consecutive case series, we enrolled patients who underwent combined phacovitrectomy with SO removal and IOL implantation and who had prior vitrectomy with SO filling due to the retinal detachment at a single centre from January 2016 to May 2023.

### 2.2 Inclusion and exclusion criteria

We included pseudophakic patients with a monofocal, non‐toric IOL with a history of combined phacovitrectomy and IOL implantation in the bag after prior vitrectomy with SO fill due to RD, without intra‐ or postoperative complications, and complete pre‐ and postoperative data of both the operated eye (OE) and FE.

We excluded patients with the following criteria: a history of corneal refractive surgery, ocular pathologies that could influence the postoperative refraction and missing pre‐ or postoperative data.

Only patients who received successful simultaneous biometric measurement of the phakic FE without a history of corneal refractive surgery, or other ocular pathologies that could influence the assessment of biometric parameters, were included. We excluded patients with a pseudophakic FE.

### 2.3 Preoperative and postoperative assessment

We used the IOLMaster 700 (Carl Zeiss Meditec AG, Jena, Germany; software V.1.50–1.90.12.05) with an integrated partial coherence interferometer (PCI) to obtain the following biometric parameters: keratometry values, horizontal corneal diameter (CD), anterior chamber depth (ACD; measured from corneal epithelium to the lens), lens thickness (LT) and AL. The used refractive index was 1.332. Measurements were performed on both eyes before vitrectomy with SO instillation. In the OE, the AL was measured under SO endotamponade using the ‘Silicone oil’ setting of the IOLMaster 700. An SO with 5000 centistokes was used in all eyes (Ophtha futur sil 5000, Pharmapur, Germany).

Objective refraction was determined by a trained technician using the Topcon KR‐800S auto kerato‐refractometer (Topcon Medical Systems, Inc., Oakland, NJ, USA). Postoperative refraction was measured at least 4 weeks after combined phacovitrectomy with SO removal. The spherical equivalent was calculated by adding the sum of sphere power with half of the cylinder power.

### 2.4 Surgical technique and intraocular lenses

In all eyes, phacoemulsification was performed through a 2.2 mm free corneal incision with IOL implantation into the capsular bag. For the removal of the SO, pars plana vitrectomy with three or four ports (23 gauge or smaller) and filling of the vitreous cavity with sulphur hexafluoride (SF_6_) gas or air were performed in one session by the same surgeon. All operations were performed by three experienced surgeons at a single centre (Department of Ophthalmology, Goethe University, Frankfurt, Germany).

In this study, five different monofocal non‐toric IOLs were implanted. The IOL calculation was performed using the suggested IOL constants for the appropriate IOL using the Barrett Universal II (BUII) formula. The different IOLs and constants for the calculation are listed in Table 1.

**TABLE 1:** Intraocular lens types, distribution and used constants.

IOL type	A‐constant BUII	*N*	%
SA60AT	118.53	4	5.1
MA60AC	119.20	4	5.1
AAB00	119.00	47	59.5
SN60WF	118.99	11	13.9
SN60AT	118.53	13	16.5

Abbreviations: %, percent; BUII, Barrett Universal II; IOL, intraocular lens; *N*, number.

### 2.5 IOL power calculation

The IOL power was calculated with the BUII formula using all required values of the FE (FE group). In addition, we used the BUII formula for the IOL calculation of the OE, using all required values of the OE with only the AL of the FE (AL‐FE group) and (2) the AL of the OE after reattachment of the retina with SO (OE group). Calculations were performed in October 2023 using the BUII Formula V1.05 provided by the Asia‐Pacific Association of Cataract and Refractive Surgeons (APACRS) (https://calc.apacrs.org/barrett_universal2105/). We only used the BUII formula in our study since it performed best in two recently published papers, both in eyes with SO endotamponade and when using the AL of the FE (Lwowski et al., 2022, 2023). The optimized A constants for the BUII formula were used as provided by the calculator of APACRS. The A constant of the ULIB database was used for AAB00, which cannot be selected in the aforementioned calculator.

For assessing the prediction error (PE) and absolute PE of the IOL power calculated with the BUII formula, we assessed the difference between the actual postoperative spherical equivalent refraction at the spectacle plane and the predicted refraction based on the implanted IOL power.

PE=Predicted refraction–Actual postoperative refraction

AbsolutePE=∣Predicted refraction–Actual postoperative refraction∣

In the interpretation of the PE, a positive value means a hyperopic, and a negative value a myopic shift.

The mean arithmetic prediction error (MPE), mean absolute prediction error (MAE), median absolute prediction error (MedAE), the maximum and minimum of these errors (range), as well as the percentage of eyes within ±0.50, ±1.00 and ±2.00 dioptres (D) deviation from the target refraction were calculated.

MedAE is less sensitive to outliers than the MAE and gives a better impression of the typical deviation in the centre of the data distribution. The MPE is helpful for identifying systematic errors.

### 2.6 Statistical analysis

In all eyes, the pseudonymized data were collected and entered manually into an Excel sheet (Version 14.7.7, Microsoft Corporation, Redmond, WA, USA). For statistical analysis, SPSS Software (Version 29.0; IBM Corporation, Armonk, NC, USA) was used. First, a Kolmogorov–Smirnov test was used to test for normal distribution. The white test was used to test for heteroscedasticity. As heteroscedasticity was only slightly present in the AL‐FE group (*p* = 0.046) and not in the other two groups (*p* > 0.05), the established statistical analysis was used for further analysis. A Friedman test, and, if significantly different, a post hoc analysis with a Wilcoxon signed‐rank test (if not parametric) or paired *t*‐test (if normally distributed), were performed to analyse differences in MPE and MedAE. To compare the percentage of PE within ±0.50 D and ± 1.00 D, the Cochrane Q test was used. *p*‐values below 0.05 were considered statistically significant. If needed, the *p*‐values were corrected using the Bonferroni correction.

The sample size estimation was performed using the G*Power 3.1 Software (Heinrich Heine University Duesseldorf, Germany). Based on a difference between the groups of 0.10 D with a standard deviation of 0.20 D, which we consider clinically relevant, at least 47 eyes were needed to reach power of at least 90% and an *α* value of 0.05.

## 3 RESULTS

In this study, 79 eyes of 79 patients matched our inclusion criteria. Fifty‐one (65%) were males. The mean age was 59.32 years ± 9.62 (Range: 24–82). The mean AL of the OE with SO fill after re‐attachment of the retina was 25.27 mm ± 1.79 mm (Range: 22.06–31.72). The mean AL of the FE was 25.11 mm ± 1.81 mm (Range: 22.05–32.08). The mean absolute difference of the AL of both eyes was 0.40 mm ± 0.50 mm (Range: 0.00–2.76 mm), mean relative difference was 0.15 mm ± 0.62 mm (Range: −1.26 – +2.76 mm). The Pearson coefficient between the AL of the FE and the OE was 0.943 (*p* < 0.001). In 23 patients, the AL was above 26.0 mm. Baseline characteristics of patients are summarized in Table 2.

**TABLE 2:** Patient demographics and baseline data of both eyes.

	Mean	Standard deviation	Minimum	Maximum
Age (years)	59.32	9.62	24	82
AL OE (mm)	25.27	1.79	22.06	31.72
AL FE (mm)	25.11	1.78	22.05	32.08
Kmean OE (D)	42.17	1.51	38.93	45.77
Kmean FE (D)	42.12	1.36	38.47	45.32
ACD OE (mm)	3.29	0.37	2.33	4.23
ACD FE (mm)	3.30	0.33	2.43	3.96
LT OE (mm)	4.45	0.44	2.92	5.62
LT FE (mm)	4.46	0.40	3.33	5.48
IOL power (D)	18.46	4.09	6	25
Postop SE (D)	−1.31	1.11	−3.75	2.00
Postop VA (log MAR)	0.36	0.44	1.7	−0.1

Abbreviations: ACD, Anterior chamber depth; AL, Axial length, D, Dioptres; FE, Fellow eye; IOL, Intraocular lens; Kmean, mean keratometry; log MAR, logarithm of the minimal angle of resolution; LT, Lens thickness; OE, operated eye; Postop SE, Postoperative spherical equivalent; VA, Visual acuity.

The implanted monofocal IOLs were AAB00 (47 eyes, 59.5%), SN60AT (13 eyes, 16.5%), SN60WF (11 eyes, 13.9%), SA60AT (4 eyes, 5.1%) and MA60AC (4 eyes, 5.1%). The mean postoperative spherical equivalent was −1.31 D ± 1.11. The mean IOL power was 18.46 D ± 4.09 (6.00–25.00 D). The follow‐up was at least one month in all patients.

**FIGURE 1:**
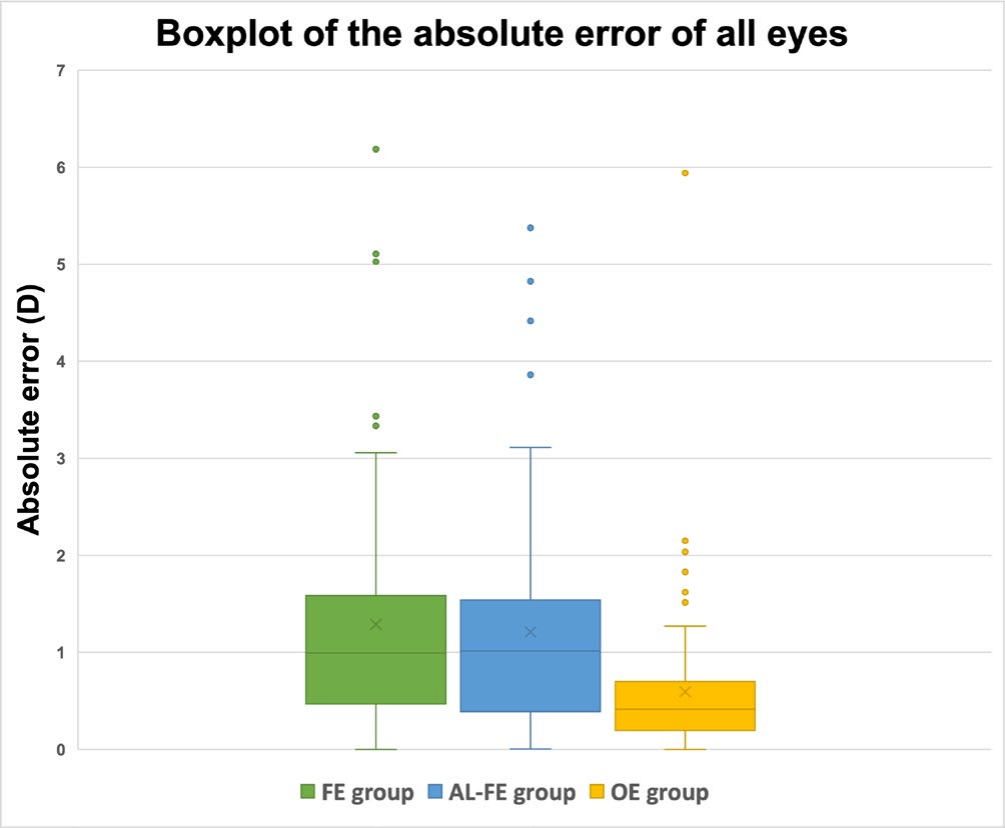
Boxplot comparing the absolute prediction error of the three groups. The box represents the lower and upper interquartile range (IQR). The whiskers represent the upper and lower 1.5‐fold IQR. Points outside the whiskers represent outliers. To calculate the prediction error, the intraocular lens calculation was performed using the Barrett Universal II formula with the biometry of the fellow eye (FE group), using only the axial length (AL) of the fellow eye (AL‐FE group) in addition to the measurable biometric parameters of the affected eye, as well as using the AL of the operated eye (OE group) after reattachment of the retina. The cross (×) represents the mean absolute error; the horizontal line (–) the median absolute error.

MedAE was lowest in the OE group (0.41 D), followed by the FE group (1.00 D) and AL‐FE group (1.02 D) (Figure 1). Using Friedman's test, the difference between the absolute errors of the three groups was statistically significant (*p* < 0.001). Post hoc analysis using the Wilcoxon signed‐rank test with Bonferroni correction yielded a statistically significant difference between the absolute error (AE) comparing the OE group and AL‐FE group (*p* < 0.001), as well as between the OE group and the FE group (*p* < 0.001). However, when comparing the differences in the AE between the FE group and the AL‐FE group, there was no statistically significant difference (*p* = 0.960). MPE, MAE and MedAE with standard deviation and range for all eyes and as a subgroup analysis of eyes that received an AAB00 IOL (*n* = 47) are shown in Table 3. No correlation was found between the AL of the OE and the absolute prediction error (Pearson coefficient = −0.091; *p* = 0.424).

**TABLE 3:** Postoperative Refractive Outcomes.

	MPE	MAE	MedAE	SD	Min	Max
All eyes (*n* = 79)						
FE group	−1.08	1.29	1.00	1.19	0.00	6.19
FE‐AL group	−0.90	1.21	1.02	1.11	0.01	5.38
OE group	−0.03	0.59	0.42	0.77	0.00	5.94
Subgroup analysis AAB00 (*n* = 47)						
FE group	−1.21	1.32	1.08	1.21	0.00	6.19
FE‐AL group	−1.07	1.25	1.04	1.09	0.01	4.83
OE group	−0.08	0.60	0.42	0.88	0.00	5.94

Abbreviations: FE, fellow eye; FE‐AL, only the axial length of the fellow eye was used in combination with the measurable biometric data of the operated eye for IOL calculation; MAE, Mean absolute error; MedAE, Median absolute error; MPE, Mean prediction error; OE, operated eye; SD, Standard deviation.

**FIGURE 2:**
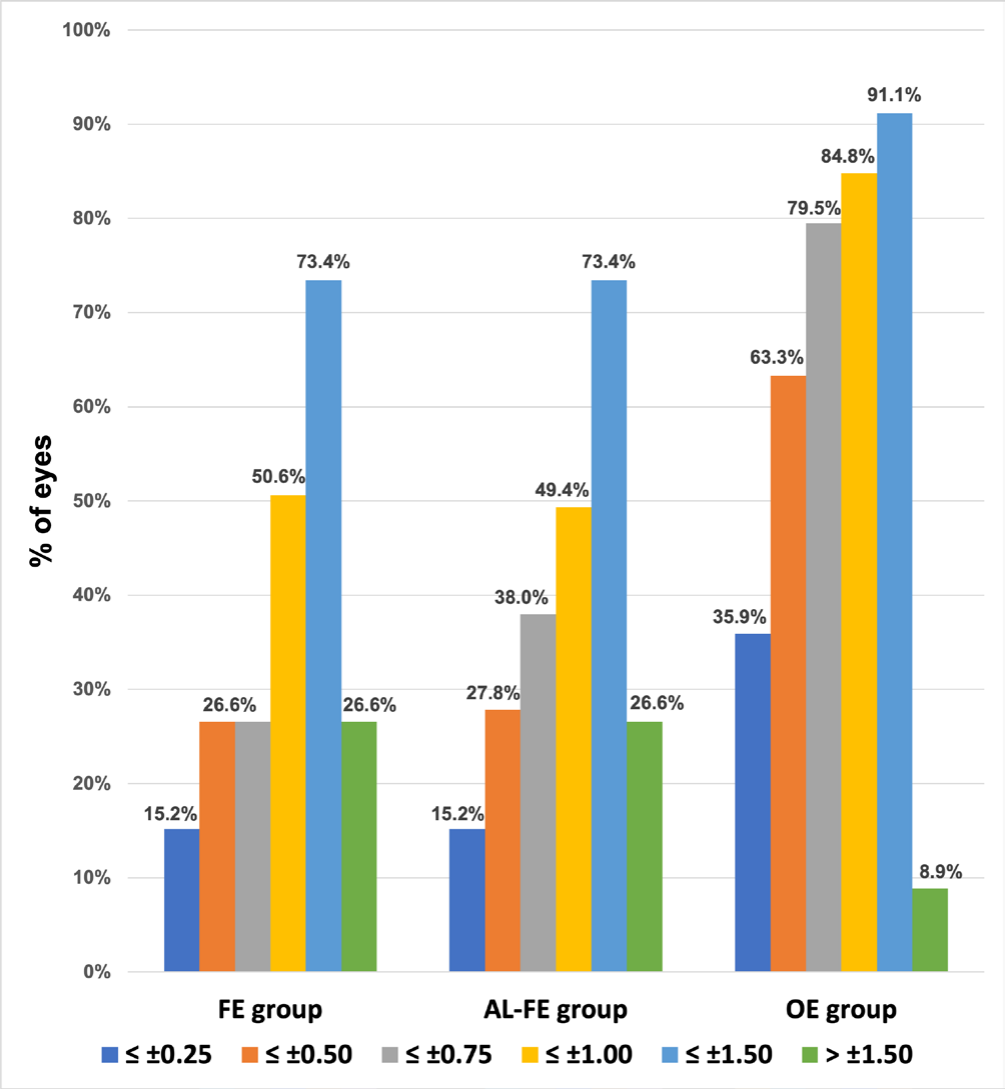
Bar chart presenting the portion of eyes within ±0.25, ±0.50, ±0.75, ±1.00, ±1.50 and >±1.50 D from target refraction. Intraocular lens calculation was performed using the Barrett Universal II formula with the biometry of the fellow eye (FE group), using only the axial length (AL) of the fellow eye (AL‐FE group) in addition to the measurable biometric parameters of the affected eye, as well as using the AL of the operated eye (OE group) after reattachment of the retina.

Considering the number of eyes within ±0.50 D of the target refraction, the OE group performed best with 50 eyes (63.3%). In contrast, both the FE and AL‐FE groups performed considerably less favourably, with 21 and 22 eyes (26.6% and 27.8%), respectively. Using the Cochrane Q test, a statistically significant difference was found between the OE and AL‐FE groups, as well as between the OE and FE groups (*p* < 0.001 each). Comparison between the AL‐FE and FE groups showed no statistically significant difference (*p* = 0.712). Figure 2 shows the number of eyes within ±0.25, ±0.50, ±0.75, ±1.00, ±1.50 and >±1.50 D of the target refraction with likewise significantly more eyes in the OE group compared to the FE and AL‐FE groups.

## 4 DISCUSSION

The AL of the eye is the most crucial parameter in IOL calculation (Langenbucher et al., 2022; Norrby, 2008). A 0.1 mm error in AL is equivalent to an error of about 0.27 D in the spectacle plane (Olsen, 2007). Therefore, high accuracy in the assessment of the AL is a necessity. For many years, in clinical practice ultrasound was the only technique to measure the length of the eye. The accuracy of the AL measurement has improved considerably with the implementation of optical biometry using PCI (Drexler et al., 1998). Despite technical progress, it is often not possible to determine the exact AL with the PCI integrated into the ocular biometer in eyes with macular‐involving retinal detachment (Liu et al., 2022). In cases of pre‐existing lens opacities or cataracts, the surgeon may use the data of the FE instead of ultrasound biometry which is known to be much less accurate (Liu et al., 2022). Some studies report sufficient results for lens power calculation and prediction of refractive outcome when biometry of the FE is used (El‐Khayat et al., 2019; Knox Cartwright et al., 2010).

In optical biometry of the eye, the refractive index of the vitreous (usually around 1.336) is crucial for the accurate measurement of the AL of the eye and for the calculation of the IOL power (Murphy & Howland, 1987). SO has a higher refractive index than the vitreous, typically around 1.40 (depending on the type of SO used) (Stefánsson et al., 1988). This difference affects the refraction of light within the eye and therefore also the biometric measurements. An increase in the refractive index due to SO leads to an apparent shortening of the AL of the eye in optical measurement methods. This is due to the fact that light rays are refracted more strongly and therefore travel a shorter optical path. If the refractive index is not correctly taken into account, this can lead to errors in the calculation of the IOL power (Figure 2).

In the present study, we investigated whether using the entire IOL calculation of the FE provides reliable results, or whether the AL of the FE in combination with the measurable biometric parameters of the affected eye induces a lower refractive error when the AL of the affected eye cannot be obtained. To the best of our knowledge, studies comparing the use of the AL of the FE for the IOL calculation of the affected eye with the entire biometry of the FE are rare.

Our results suggest that using the biometry of the FE (MedAE 1.00 D) for IOL calculations for macular‐involving RD is as accurate as using only the AL of the FE in combination with the measurable biometric parameters of the OE (MedAE 1.02 D). In our study, every second eye was within 1.00 D of its predicted refractive outcome. This is supported by the fact that the symmetry between both eyes is given and showed good agreement if performed with the advice used in our study (Albarrán‐Diego et al., 2022). For AL shorter than 24 mm, good agreement was shown, too. Only for eyes with an AL >24 mm, moderate agreement was seen in the interocular differences for AL measurements. Kristianslund et al. (2022) investigated the use of biometric measurements of the FE to compensate for missing values. Since the assumption that both eyes of a person are symmetrical and nearly similar is not always true, the IOL power was calculated using the biometric parameters of the FE. The results of 500 patients (1000 phakic eyes) showed that the use of the calculated lens power of the FE resulted in a mean difference of 0.79 D and a difference of more than 1.0 D in 23% of patients. Our study population included eyes with an RRD that showed a tendency to be myopic (mean AL of the fellow eye was 25.11 mm ± 1.81 mm). This could be a reason why in our study the difference when the IOL power of the FE was used was slightly larger than in the study by Kristianslund et al. (2022) which included 500 adult patients scheduled for cataract surgery.

Comparing this with the outcome achieved when using the biometry of the OE after reattachment of the retina with SO fill, the IOL power predictability error is statistically and clinically significantly lower using a two‐step procedure (MedAE 0.41 D) (*p* < 0.001). Furthermore, far more patients achieve an outcome within ±1.00 D of prediction (84.8%).

In order to avoid postoperative anisometropia, it may be useful to ask the patient preoperatively whether there are differences between the refractive values of the two eyes and to rule out preoperative anisometropia. In our opinion, it is not advisable to use the biometric values of the fellow eye if there are major differences between the eyes, in order to avoid refractive surprises. We agreed with El‐Khayat et al. (2019) who determined that it is possible to use the IOL calculation of the partner eye if the refraction values of both eyes are quite similar. Manual entry of the AL into the biometry device is, based on the results of the present study, not superior to using the entire IOL calculation of the FE. A definite solution for this could be an intraoperative AL measurement after reattachment of the retina (Moussa et al., 2021). However, as long as this is not possible, the results of the FE show a higher predictability compared to the calculation with an AL assessed with the macula detached, but as we could show in this and the previous publication, still a significantly worse result compared to the calculation with the AL in SO‐filled eyes (Lwowski et al., 2023).

In addition to preoperative anisometropia, conditions that may preclude the use of biometry of the FE include corneal disease, poor fixation, significant media opacity, and cataract and macular elevation/oedema.

The limitations of our study include the retrospective setting and the fact that different IOLs were included. However, the authors believe that the sample size is sufficient for this purpose. In addition, the IOL calculation of the FE and the use of the AL of the FE were only performed theoretically, without corresponding implantation of the IOL in the affected eye. To best reflect the daily practice of most clinicians, we did not optimize the constants for the IOL calculation and decided to use proven constants. In our opinion, it was not necessary to optimize the constants as biometric data of the FE was used. Based on the recommendations of Hoffer and Savini for reporting IOL calculation results, it shows that optimization of IOL constants is not indicated in highly specific and rare cases, which in our view would also apply to our study (Hoffer & Savini, 2021).

However, when calculating lens replacement, accurate predictability can prevent anisometropic problems that could consecutively lead to lens surgery in the healthy eye, which could result in surgical risks such as retinal detachment or infection.

To conclude, our results indicate no clinically relevant difference between using the entire IOL calculation of the FE versus using only the AL of the FE in addition to the measurable biometric parameters of the affected eye for the determination of the IOL power. However, a two‐step procedure using the AL of the OE after reattachment of the retina is still highly recommended for the best possible calculation of the IOL power.

## FUNDING INFORMATION

No funding was received for this study.

## CONFLICT OF INTEREST STATEMENT

T. Kohnen: Consultant and Research for Alcon/Novartis, J&J, Lensgen, Oculentis, Oculus, Presbia, Schwind, Zeiss. Consultant for Allergan, Bausch & Lomb, Geuder, Med Update, Santen, Staar, Thieme, Ziemer. All other authors have no financial interests to disclose.

## ETHICS STATEMENT

This study includes human participants and was approved by the ethics committee of the Goethe University Frankfurt (Approval number: 2022–899). Before participating in the study, the participants gave their informed consent after being informed in detail.

## Data Availability

All data generated or analysed during this study are included in this article. Further enquiries can be directed to the corresponding author.
